# A food-medicine homology formulation ameliorates atherosclerosis by attenuating dyslipidemia and inflammation via the PI3K/Akt/NF-κB pathway

**DOI:** 10.3389/fphar.2025.1710585

**Published:** 2025-12-04

**Authors:** Yadong Zheng, Kaili Chen, Shuo Zhang, Junsong Jing, Zhihao Zhou, Junfeng Lu, Christian Holscher, Wenlong Chen, Liguo Li

**Affiliations:** 1 Institute of Rehabilitation Medicine, Henan Academy of Innovations in Medical Science, Zhengzhou, China; 2 Department of Nephropathy, The First Affiliated Hospital of Henan University of Chinese Medicine, Zhengzhou, China; 3 Henan Qingmaitong Biotechnology Co., Ltd., Zhengzhou, China; 4 Henan Airport Investment Big Health Industry Group Co., Ltd., Zhengzhou, China

**Keywords:** food-medicine homology, atherosclerosis, cardiovascular disease, inflammation, PI3K

## Abstract

**Background:**

Atherosclerosis (AS), a chronic inflammatory condition of the vasculature, is a major contributor to cardiovascular morbidity. Yaoshi Tongyuan Tablet (YTT) is a food-medicine homology (FMH) formulation containing *Panax Ginseng*, *Radix Puerariae*, *Sophora Japonica*, *Ginkgo Semen*, *Persicae Semen*, *Ziziphi Spinosae Semen*, and *Crataegus Pinnatifida*, which shows promise for AS management; however, its mechanistic basis remains poorly defined. This study aims to explore the protective roles and mechanistic foundations of YTT in preventing and treating AS.

**Methods:**

A combination of network pharmacology, ultra-performance liquid chromatography coupled with Q Exactive Orbitrap mass spectrometry (UPLC-QE-MS), and molecular docking was employed to predict potential bioactive compounds and their molecular targets. ApoE^−/−^ mice on a high-fat diet were used to model AS and were treated with low (250 mg/kg), medium (500 mg/kg), and high (1,000 mg/kg) doses of YTT for 4 weeks. Therapeutic efficacy and underlying molecular mechanisms were evaluated through biochemical assays, histopathological analysis, and Western blotting.

**Results:**

Integrated analyses revealed kaempferol, isorhamnetin, and quercetin as central bioactive molecules acting on AKT1, a key node within the PI3K/Akt signaling cascade. *In vivo*, YTT treatment markedly curbed weight gain, ameliorated dyslipidemia, reduced systemic inflammation, and diminished atherosclerotic plaque load, alongside promoting plaque stabilization. At the molecular level, YTT substantially inhibited activation of the PI3K/Akt axis and phosphorylation of NF-κB, leading to lowered secretion of pro-inflammatory cytokines.

**Conclusion:**

YTT ameliorates atherosclerosis by counteracting dyslipidemia and inflammation, primarily through modulation of the PI3K/Akt/NF-κB pathway. This study offers novel integrative insights into the anti-atherogenic properties of YTT and pinpoint crucial bioactive constituents worthy of further pharmacological investigation.

## 1 Introduction

Atherosclerosis (AS) constitutes a fundamental pathological process underlying coronary artery disease, ischemic stroke, and peripheral arterial diseases, representing a substantial worldwide public health challenge (Mensah et al., 2023). Despite the broad application of conventional pharmacotherapies—including statins—in clinical settings, these interventions frequently fall short of comprehensively targeting the multifactorial etiology of AS and can be accompanied by undesirable side effects (Ramkumar et al., 2016). Adjustments in lifestyle, especially through diet (Riccardi et al., 2022) and nutritional approaches (Vesnina et al., 2022), are increasingly regarded as critical measures for preventing AS (Huang et al., 2023). Within this framework, food-medicine homology (FMH)—acknowledged both as edible ingredients and agents of traditional medicine (Wang et al., 2025)—have attracted growing interest owing to their favorable safety characteristics and health-enhancing benefits (Huang et al., 2023). Consequently, there is a compelling need to identify FMH-derived substances capable of regulating pivotal pathogenic mechanisms in AS.

Yaoshi Tongyuan Tablet (YTT), a nutritional supplement formulated based on FMH, comprises seven herbal components: *Panax Ginseng*, *Radix Puerariae*, *Sophora Japonica*, *Ginkgo Semen*, *Persicae Semen*, *Ziziphi Spinosae Semen*, and *Crataegus Pinnatifida*. Existing research indicates that the bioactive constituents in YTT confer protective effects on the cardiovascular system. As an example, ginsenoside Rb1—a compound derived from *Panax Ginseng*—suppresses both NF-κB and MAPK signaling pathways. This action mitigates endothelial injury through inhibition of Lectin-like oxLDL receptor-1 (LOX-1) and reduction of NF-κB-driven inflammatory responses (Gao et al., 2020). Another major ingredient, puerarin from *Radix Puerariae*, modulates gut microbial composition and decreases trimethylamine synthesis, leading to reduced plasma levels of trimethylamine-N-oxide (TMAO), a metabolite known to promote atherosclerosis. Notably, these benefits were observed in patients with carotid plaques after only 1 week of oral intake (Li Z. et al., 2024). Additionally, quercetin, isolated from *Sophora Japonica*, obstructs calcium influx mediated by Piezo1 and inhibits subsequent activation of the NF-κB/NLRP3 inflammasome pathway. Consequently, it reduces secretion of IL-1β and other pro-inflammatory cytokines, thereby alleviating vascular inflammation and slowing the progression of AS (Wang et al., 2024). Nevertheless, a thorough profiling of YTT’s nutritional makeup and its detailed mechanisms in preventing AS has not yet been completely established.

Network pharmacology, a discipline rooted in systems biology, has become an invaluable framework for elucidating intricate interplays among bioactive molecules and pathological targets through integrated analysis of compound-target-pathway networks (Deng et al., 2025; Li H. et al., 2024). Complementing this, molecular docking approaches—informed by compound characterization via UPLC-Q Exactive Orbitrap-MS (UPLC-QE-MS)—can experimentally assess interactions between ligands and proteins by computational simulation of binding affinities (Abulaiti et al., 2024; Chen et al., 2024), thereby connecting theoretical network models with empirical evidence at the molecular scale. In this investigation, we adopted a comprehensive strategy that integrated network pharmacology, UPLC-QE-MS analysis, molecular docking, and *in vivo* validation in an ApoE^−/−^ mouse model of AS induced by a high-fat diet (HFD). The objective was to elucidate the primary active components of YTT, along with their corresponding molecular targets and associated signaling mechanisms, with a specific emphasis on the PI3K/Akt/NF-κB pathway, which is associated with inflammation and lipid metabolism regulation in AS. These findings offer critical perspectives on the potential use of FMH-based formulations as nutritional interventions for the prevention and treatment of AS.

## 2 Materials and methods

### 2.1 Chemicals and reagents

To assess lipid profiles and inflammatory markers, a series of assay kits were acquired. Total cholesterol (TC, S03042), high-density lipoprotein cholesterol (HDL-C, S03025), low-density lipoprotein cholesterol (LDL-C, S03029), triglycerides (TG, S03027), alanine aminotransferase (ALT, S03030), and aspartate aminotransferase (AST, S03040) kits were sourced from Rayto Life and Analytical Co., Ltd. (Shenzhen, China). ELISA kits for tumor necrosis factor-α (TNF-α, 88–7,324), interleukin-1β (IL-1β, 88–7,013), interleukin-6 (IL-6, 88–7,064), and interleukin-10 (IL-10, 88–7,105) were procured from Thermo Fisher Scientific (Waltham, MA, United States). Staining reagents including Oil Red O (G1015), hematoxylin and eosin (H&E, G1076), and Masson’s trichrome staining solution (G1006) were supplied by Servicebio (Wuhan, China). Atorvastatin (Lipitor) was provided by Pfizer Inc. (New York, United States). Primary antibodies targeting PI3K (ab151549) and AKT (ab8805) were obtained from Abcam (Cambridge, United Kingdom), and anti-p-PI3K (BS-5570R) was from BIOSS (Beijing, China). Additional antibodies—p-AKT (GB150002), TNF-α (GB11188), IL-6 (GB11117), IL-1β (GB11113), IκBα (GB13212-1), p-IκBα (GB15212), and p65 (GB11997)—were purchased from Servicebio. Anti-p-p65 (AP1294) was acquired from ABclonal (Wuhan, China). The HRP-conjugated goat anti-rabbit IgG secondary antibody (GB23303) and the β-actin antibody (GB15003), which served as the loading control, were also obtained from Servicebio. HFD (D12108C, 40% fat, 1.25% cholesterol) was purchased from Medicience (Jiangsu, China).

### 2.2 Identification of bioactive compounds and target prediction for YTT

To identify the bioactive compounds in seven FMH components—*Panax Ginseng* (Renshen, Ginseng), *Sophora Japonica* (Huaihua, Sophora Flower), *Radix Puerariae* (Gegen, Kudzu Root), *Ginkgo Semen* (Baiguo, Ginkgo Seed), *Ziziphi Spinosae Semen* (Suanzaoren, Jujube Seed), and *Persicae Semen* (Taoren, Peach Kernel), we extracted candidate compounds from the Traditional Chinese Medicine Systems Pharmacology Database (TCMSP, https://tcmsp-e.com/). Additionally, compounds from Crataegus pinnatifida (Shanzha, Hawthorn Fruit; HERB004945) were acquired via the HERB platform (http://herb.ac.cn/). Following established pharmacokinetic guidelines (Liu et al., 2013), we applied an initial filtering step based on oral bioavailability (OB ≥ 30%) and drug-likeness (DL ≥ 0.18). The Canonical SMILES strings of these filtered compounds were subsequently retrieved from PubChem (https://pubchem.ncbi.nlm.nih.gov/) and submitted to the SwissADME tool (http://www.swissadme.ch/) for further evaluation of gastrointestinal (GI) absorption and drug-likeness characteristics. Subsequent prediction of potential targets for the chosen compounds was conducted via the SwissTargetPrediction platform (http://www.swisstargetprediction.ch/) by inputting their SMILES notations. The resulting target gene nomenclature was then normalized in the UniProt database (https://www.uniprot.org/), with all queries limited to the species *Homo sapiens*.

### 2.3 Identification of atherosclerosis-associated targets

To identify targets associated with atherosclerosis, we conducted a systematic search using the keyword “atherosclerosis” across multiple disease databases, including GeneCards (https://www.genecards.org/), OMIM (https://www.omim.org/), and DisGeNET (https://www.disgenet.org/). The retrieval process was restricted to targets explicitly annotated for *Homo sapiens*. All acquired gene symbols underwent normalization via the UniProt database to ensure nomenclature consistency. Following curation, the datasets from each source were combined and deduplicated to yield a unified list of AS-related targets.

### 2.4 Protein-protein interaction (PPI) network construction and analysis

To construct the protein interaction network, common targets identified in Sections 2.2, 2.3 were analyzed using the STRING database (https://string-db.org/), with the species parameter specified as *Homo sapiens*. The resulting interactome was imported into Cytoscape (version 3.10.3) for visualization and further examined with the CentiScaPe 2.2 plugin to assess network topology. Using this tool, we computed three centrality metrics—degree centrality, closeness centrality, and betweenness centrality. Targets that scored above the median values across all three parameters were classified as core targets. The network integrating FMH substances, bioactive compounds, and their predicted targets was built with Cytoscape (v3.10.3). To indicate topological relevance, node dimensions and coloring were assigned based on degree values. To generate a holistic “substance-compound-target-pathway” network, we integrated data on seven FMH substances, bioactive compounds, predicted targets, and enriched pathways related to YTT, and imported these into Cytoscape. Within this network, nodes were distinguished by specific shapes and colors to represent distinct categories. Connections between nodes were depicted as edges, providing a clear visual mapping of their interrelationships.

### 2.5 Functional Enrichment analysis via GO and KEGG

To elucidate the functional implications of the common targets, we performed comprehensive Gene Ontology (GO) and Kyoto Encyclopedia of Genes and Genomes (KEGG) pathway enrichment analyses using the DAVID bioinformatics platform (version 6.8; https://david.ncifcrf.gov/). Enrichment results were systematically ranked according to gene count. Functional annotations were evaluated across the three principal GO domains: biological processes (BP), molecular functions (MF), and cellular components (CC). The ten most significantly enriched terms from the GO analysis were selected for visualization in a bar plot. Additionally, the top twenty enriched KEGG pathways were graphically represented using a bubble chart, which integrates statistical significance and magnitude of enrichment.

### 2.6 Analysis of YTT components using UPLC-QE-MS

For the identification of chemical constituents in YTT, 0.1 g of powdered sample was processed with 1,200 μL of 80% methanol in the presence of grinding beads. The mixture was homogenized by grinding for 5 min, followed by vortexing for 10 min and subsequent centrifugation at 13,000 rpm for an additional 10 min. The resulting supernatant was carefully collected and subjected to UPLC-QE-MS analysis. Chromatographic separation was achieved using an AQ-C18 column (150 × 2.1 mm, 1.8 μm, Welch) with a binary mobile phase composed of 0.1% formic acid in water (A) and methanol (B), delivered at a constant flow rate of 0.30 mL/min. The column temperature was maintained at 35 °C, while the autosampler was kept at 10 °C; each injection volume was 5.0 μL. Mass spectrometric detection was performed on a Q Exactive high-resolution instrument (Thermo Fisher Scientific) featuring an electrospray ionization (ESI) source operated in both positive and negative modes, scanning across a mass range of m/z 100–1,500. Key ESI parameters included a spray voltage of 3.2 kV and capillary temperature of 300 °C. High-purity argon (≥99.999%) served as the collision gas, with normalized collision energies (NCE) set at 30, 40, and 60. Nitrogen (≥99.999%) was employed as both sheath gas (40 Arb) and auxiliary gas (15 Arb), heated to 350 °C. Total data acquisition time spanned 30 min. Raw data were analyzed using Compound Discoverer 3.3 (Thermo Fisher), and compounds were identified by querying the mzCloud database.

### 2.7 Molecular docking analysis

Based on the core targets and KEGG enrichment findings, the PI3K-Akt signaling pathway was chosen for in-depth investigation. Key targets and bioactive compounds associated with this pathway were pinpointed through the previously established “substance-compound-target-pathway” network and further corroborated by UPLC-QE-MS results. The three-dimensional structure of AKT1 was acquired from the RCSB Protein Data Bank (https://www.rcsb.org/), from which all water molecules and non-essential ligands were eliminated. Preprocessing of the protein—including hydrogen addition, charge assignment, and rotatable bond identification—was conducted using AutoDock Tools. Similarly, 3D structures of the active compounds, previously identified via UPLC-QE-MS, were retrieved from PubChem (https://pubchem.ncbi.nlm.nih.gov/) and prepared with the same toolkit, incorporating charge calculation and rotatable bond specification. A docking grid box was configured according to the predicted or documented active site of each target protein. Docking simulations were carried out with AutoDock Vina, and the conformation exhibiting the lowest binding free energy for each protein-ligand pairing was selected as the most favorable outcome. Final docking interactions were visualized and interpreted using PyMOL.

### 2.8 Animals and experimental design

All animal experimental procedures were conducted in strict compliance with the National Institutes of Health (NIH) guidelines (Guide for the Care and Use of Laboratory Animals, 1996) and received formal approval from the Animal Ethics Committee of the Servicebio Laboratory Animal Center (Approval Code: 2024203). Male C57BL/6J mice and male ApoE^−/−^ mice with a C57BL/6 genetic background, aged 7 weeks (body weight 20 ± 2.0 g) and confirmed to be specific pathogen-free (SPF), were supplied by Beijing Vital River Laboratory Animal Technology Co., Ltd. (Beijing, China). Following a one-week acclimatization period under SPF housing conditions, the ApoE^−/−^ mice were subjected to an atherogenic high-fat diet (HFD, D12108C; Medicience, Jiangsu, China), containing 40% fat and 1.25% cholesterol, to induce atherosclerotic lesions. In parallel, the wild-type C57BL/6J control mice continued to receive a standard laboratory chow diet. This dietary regimen was maintained for a total duration of 12 weeks to ensure robust development of the atherosclerosis model prior to pharmacological intervention.

### 2.9 Animal treatment and specimen harvest

Following the successful induction of the model, mice were allocated randomly into six cohorts (n = 8 per group) according to their body weight. Each intervention was delivered once per day in the morning (between 9:00 a.m. and 10:00 a.m.) through oral gavage over a period of 4 weeks. The experimental design comprised the following groups: (i) Control (CON): C57BL/6J mice maintained on a standard diet and receiving distilled water; (ii) Model (MOD): ApoE^−/−^ mice fed an HFD and given distilled water; (iii) YTT low dose (L): ApoE^−/−^ mice fed an HFD and administered YTT (250 mg/kg/day); (iv) YTT medium dose (M): ApoE^−/−^ mice fed an HFD and administered YTT (500 mg/kg/day); (v) YTT high dose (H): ApoE^−/−^ mice fed an HFD and administered YTT (1,000 mg/kg/day); (vi) Positive control (ATV): ApoE^−/−^ mice fed an HFD and administered atorvastatin (Lipitor) at a dosage of 10 mg/kg/day (Lin et al., 2024). YTT dosages were determined by human-to-mouse dose conversion based on body surface area, formulated at a ratio of 1:2:4 to represent low, medium, and high dose equivalents. Throughout the experimental timeline, body weight was measured on a weekly basis. After intervention, animals were anesthetized and subsequently sacrificed. Blood was drawn into both heparinized and plain tubes, and after centrifugation, plasma and serum aliquots were isolated and cryopreserved at −80 °C for further assessment. Cardiac perfusion with ice-cold phosphate-buffered saline (PBS) was then conducted through the left ventricle. The entire aorta and heart were carefully excised. Tissue samples were either immersion-fixed in 4% paraformaldehyde for histological examination or frozen in liquid nitrogen and maintained at −80 °C for subsequent molecular assays.

### 2.10 Analysis of serum lipid profiles and inflammatory cytokine

Quantification of serum TG, TC, LDL-C, HDL-C, ALT, and AST was conducted using a fully automated biochemical analyzer (Chemray 240/420/800; Rayto, China) with corresponding commercially available kits, in accordance with the protocols provided by the manufacturer. Serum concentrations of TNF-α, IL-1β, IL-6, and IL-10 were quantified using enzyme-linked immunosorbent assay (ELISA) kits in accordance with the manufacturer’s guidelines. Absorbance measurements were taken at 450 nm with a microplate reader (Epoch, BioTek, United States), and cytokine levels were determined through interpolation from standard curves.

### 2.11 Analysis of atherosclerotic lesions

Aortic tissues were isolated and meticulously cleansed under a stereomicroscope. Following fixation with 4% paraformaldehyde, longitudinal aortic sections were subjected to Oil Red O staining, imaged with a Leica microscopy system (Germany), and quantitatively assessed using ImageJ software. To evaluate lesions in the aortic root, samples underwent sucrose gradient dehydration, were embedded in optimal cutting temperature compound (OCT), and cryosectioned prior to Oil Red O staining for visualization of lipid deposition. Parallel sections from the same aortic root region were additionally embedded in paraffin, sliced into 5–6 μm sections, and stained with hematoxylin and eosin as well as Masson’s trichrome following established protocols. Plaque area, lipid accumulation, and collagen content were measured and analyzed using ImageJ.

### 2.12 Western blot analysis

Protein extraction from aortic tissues was performed using RIPA lysis buffer containing phenylmethylsulfonyl fluoride (PMSF). Protein concentration was quantified with the bicinchoninic acid (BCA) assay. Equal quantities of protein were resolved by SDS-PAGE and electrophoretically transferred to PVDF membranes. The membranes were incubated with specific primary antibodies, followed by horseradish peroxidase (HRP)-conjugated secondary antibodies. Protein bands were detected by enhanced chemiluminescence (ECL) and documented using a Bio-Rad imaging system. Quantification of band intensity was carried out with ImageJ software. β-Actin was used as an internal control for normalization.

### 2.13 Statistical analysis

Data are presented as mean ± SD derived from a minimum of three independent experiments. GraphPad Prism 10 was employed to conduct statistical analyses. Comparisons among groups were made using ANOVA with Tukey’s *post hoc* test. Differences were considered statistically significant at P < 0.05.

## 3 Results

### 3.1 Composition of YTT and putative targets for atherosclerosis

**FIGURE 1:**
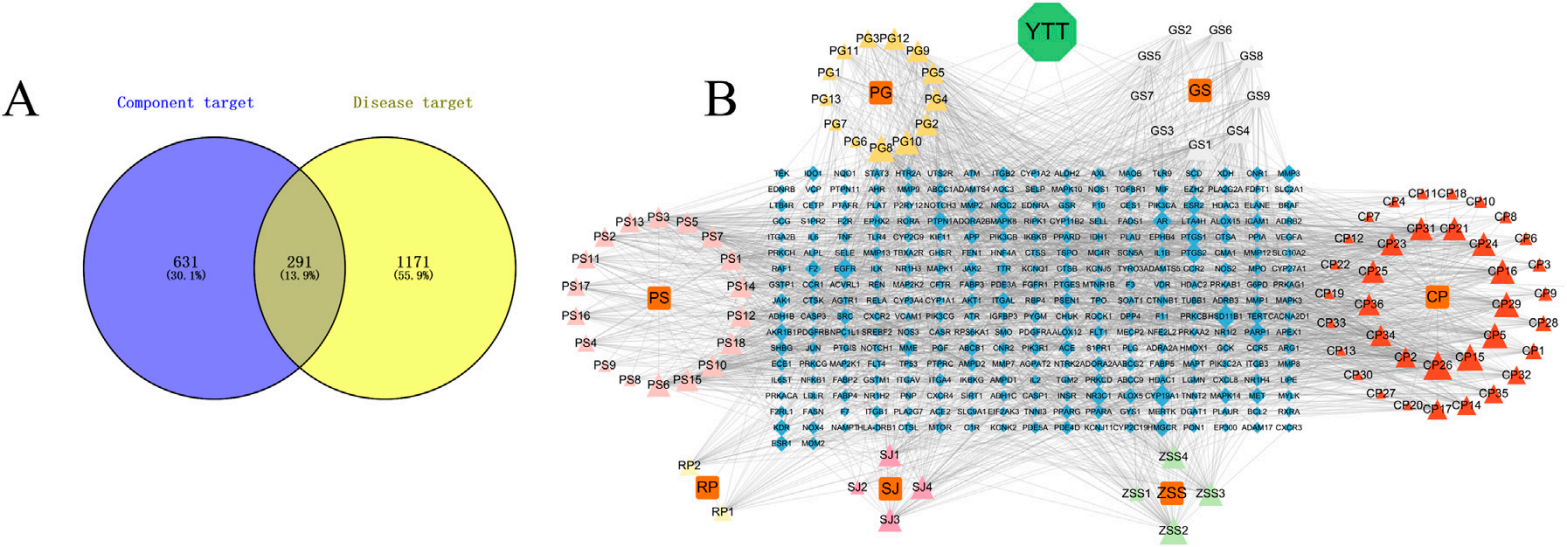
Network Pharmacology-Based Exploration of YTT for AS Intervention. **(A)** Venn diagram representing the intersection of targets associated with YTT and AS-associated targets. **(B)** Network of substance-compound-target, depicting connections among herbal ingredients, bioactive compounds, and putative targets. PG, Panax Ginseng; GS, Ginkgo Semen; PS, Persicae Semen; CP, Crataegus Pinnatifida; RP, Radix Puerariae; SJ, Sophora Japonica; ZSS, Ziziphi Spinosae Semen.

Initial screening based on OB ≥ 30% and DL ≥ 0.18, followed by the elimination of duplicate entries, yielded 179 bioactive compounds derived from the TCMSP and HERB databases corresponding to the 7 herbal substances of YTT. Subsequent refinement using criteria including high GI and at least two “yes” ratings in drug-likeness evaluation resulted in the retention of 89 compounds, such as ginsenoside Rh4, ginkgolide B, quercetin, and formononetin. The candidate targets associated with each herbal conpounds were as follows: Crataegus Pinnatifida (596), Panax Ginseng (520), Ginkgo Semen (360), Ziziphi Spinosae Semen (291), Persicae Semen (230), Sophora Japonica (156), and Radix Puerariae (67) culminating in 2,220 potential targets. Removal of 1,298 duplicates yielded 922 unique targets linked to the compounds. Regarding disease-related targets, queries of the GeneCards, OMIM, and DisGeNET databases returned 5,649, 3, and 30 AS-associated genes, respectively. After deduplication and application of a median-based score threshold, 1,642 non-redundant AS-associated targets were maintained. Taking the intersection between YTT- (n = 922) and AS-associated targets (n = 1,642) revealed 291 common genes (Figure 1A). A network illustrating “substance-compound-target” interactions was subsequently generated with Cytoscape 3.10.3 to depict relationships among herbal components, bioactive compounds, and their predicted targets (Figure 1B).

### 3.2 Protein-protein interaction network

**FIGURE 2:**
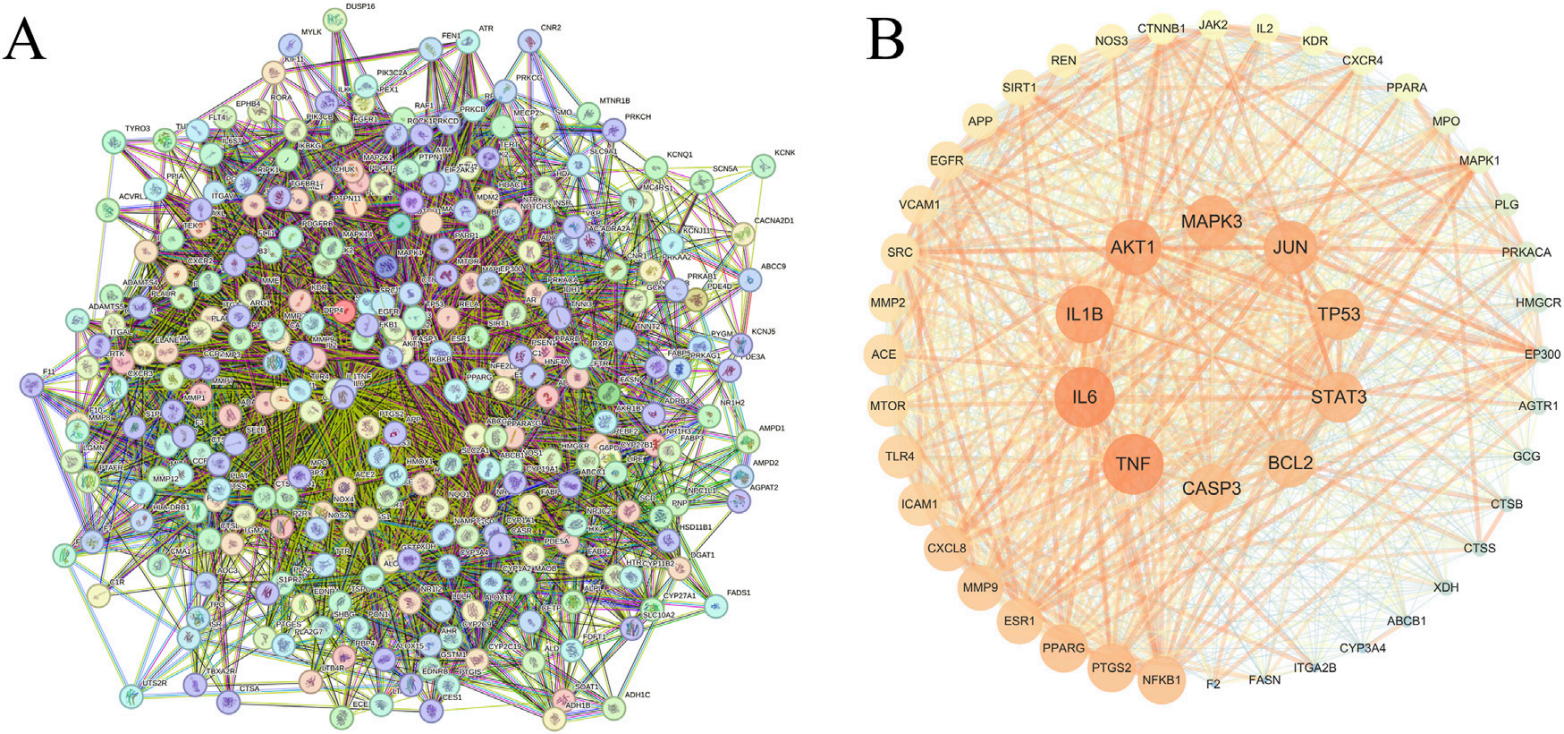
Protein-Protein Interaction Network and Core Target Identification of YTT in AS Intervention. **(A)** Protein-protein interaction (PPI) network comprising 291 common targets between YTT- and AS-associated targets. **(B)** Top 50 core targets filtered by degree centrality, betweenness centrality, and closeness centrality, visualized using Cytoscape. Node size corresponds to degree centrality values; edge thickness represents the strength of molecular interactions.

To elucidate functional relationships among the 291 common targets, a PPI network was generated using the STRING database and rendered in Cytoscape 3.10.3 (Figure 2A). The median degree centrality, closeness centrality, and betweenness centrality values were 42.25, 286.87, and 0.00176, respectively. Applying these cutoff values, 50 core targets surpassing all three median metrics were identified (Figure 2B). Among these, the top 10 hub genes—TNF, IL6, IL1B, AKT1, MAPK3, JUN, TP53, STAT3, BCL2, and CASP3—are presented with detailed annotations in Supplementary Table S1.

### 3.3 Functional enrichment analysis of common targets between YTT and atherosclerosis

**FIGURE 3:**
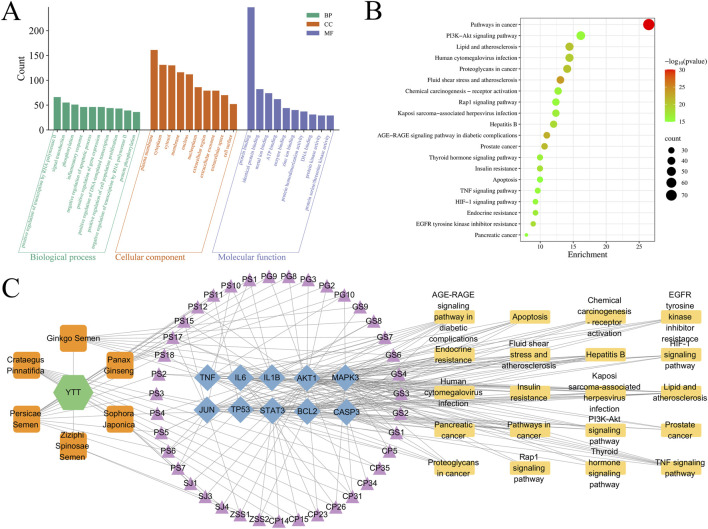
Functional Enrichment and Integrated Network Analysis of YTT in AS Treatment. **(A)** Gene Ontology (GO) enrichment results across Biological Process, Cellular Component, and Molecular Function categories. **(B)** Significantly enriched pathways identified through Kyoto Encyclopedia of Genes and Genomes (KEGG) analysis. **(C)** Substance-Compound-Target-Pathway multilevel network illustrating systematic mechanistic relationships.

To elucidate the potential mechanisms through which YTT exerts its effects against AS, GO and KEGG enrichment analyses were conducted using the DAVID database. GO analysis revealed that the common targets were significantly enriched in BP including inflammatory response and protein phosphorylation. In CC category, these targets were mainly localized to the plasma membrane, cytosol, and nucleoplasm. For MF, the most significantly enriched terms were protein kinase activity and protein serine/threonine kinase activity (Figure 3A). KEGG pathway analysis further demonstrated involvement of these targets in key AS-associated pathways, including the PI3K-Akt signaling pathway, TNF signaling pathway, lipid and atherosclerosis, and apoptosis (Figure 3B). Utilizing the core targets and significantly enriched pathways, an integrative “substance-compound-target-pathway” network was built with Cytoscape 3.10.3, incorporating FMH substances, bioactive compounds, core AS-related targets, and relevant KEGG pathways (Figure 3C). These results indicate that YTT could mitigate AS through regulation of key inflammatory signaling pathways, notably the PI3K-Akt and TNF axes, and by affecting central inflammatory mediators such as TNF, IL6, and IL1B. Given the pivotal role of the PI3K-Akt and TNF pathways in triggering and sustaining inflammatory responses, particularly via downstream NF-κB activation, which controls the expression of multiple pro-inflammatory cytokines, we further examined the binding interactions between bioactive compounds and AKT1.

### 3.4 Identification of AKT1-targeting compounds in YTT

**FIGURE 4:**
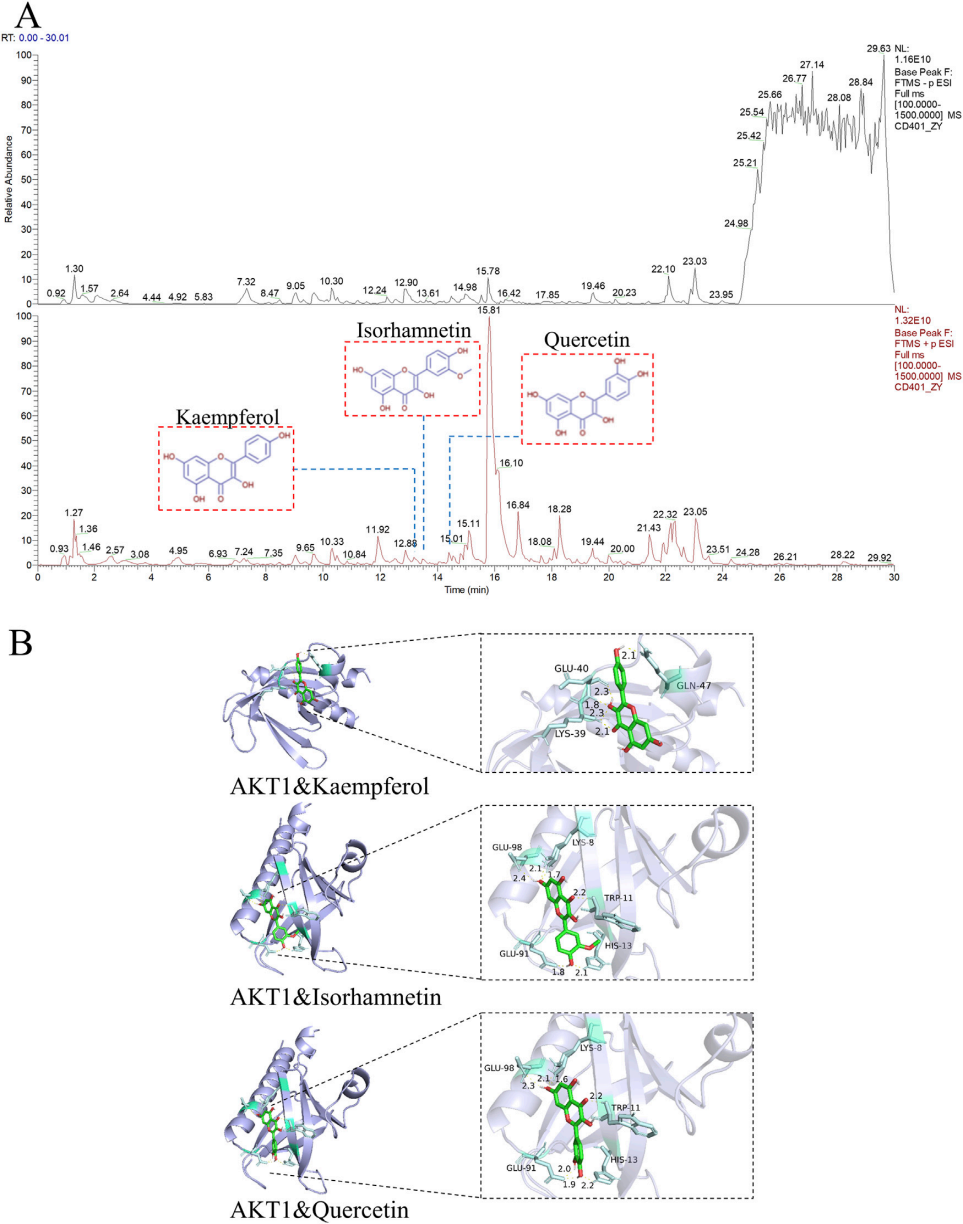
Identification of AKT1-Targeting Constituents in YTT and Computational Docking Validation. **(A)** UPLC-QE-MS total ion chromatograms of YTT. The upper trace (black) corresponds to negative ion mode detection; the lower trace (red) indicates positive ion mode. **(B)** Molecular docking simulations illustrating the binding conformations of kaempferol, isorhamnetin, and quercetin (ligands) within the AKT1 binding pocket, depicting the predicted ligand-receptor binding modes and specific residue interactions.

From the integrated substance-compound-target-pathway network, 13 candidate bioactive compounds within YTT were identified as potential interactors with the pivotal target AKT1. These included kaempferol, girinimbin, aposiopolamine, isorhamnetin, among others (Supplementary Table S2). To confirm the actual presence of these predicted compounds, UPLC-QE-MS analysis was conducted. After deduplication of entries originating from multiple substance sources, three compounds were conclusively detected in YTT, kaempferol match score 99.6, isorhamnetin match score 98.4, and quercetin match score 98.2 (Figure 4A). These findings lend experimental support to the potential functional relevance of these compounds in AKT1-mediated mechanisms and imply a possible contribution to the anti-atherosclerotic effects of YTT.

To explore the binding characteristics of key YTT-derived bioactive compounds to AKT1, molecular docking simulations were performed. The 3-dimensional structure of the AKT1 protein was acquired from the PDB database, while the structures of kaempferol, isorhamnetin, and quercetin were obtained from PubChem. Binding affinities were evaluated and yielded the following free energy values: kaempferol, −6.67 kcal/mol; isorhamnetin, −6.38 kcal/mol; and quercetin, −6.18 kcal/mol. Each compound displayed negative binding energy values, indicative of spontaneous and thermodynamically favorable binding with AKT1. Notably, all computed energies were below −5.0 kcal/mol, implying moderate binding affinity. Kaempferol exhibited the most favorable docking profile among the three ligands. The binding modes and specific residue interactions between AKT1 and each compound were visualized using PyMOL (Figure 4B). Together, these computational findings support the notion that active compounds of YTT may functionally engage with AKT1, a target relevant to anti-atherosclerotic mechanisms.

### 3.5 YTT ameliorates dyslipidemia and systemic inflammation in atherosclerosis

**FIGURE 5:**
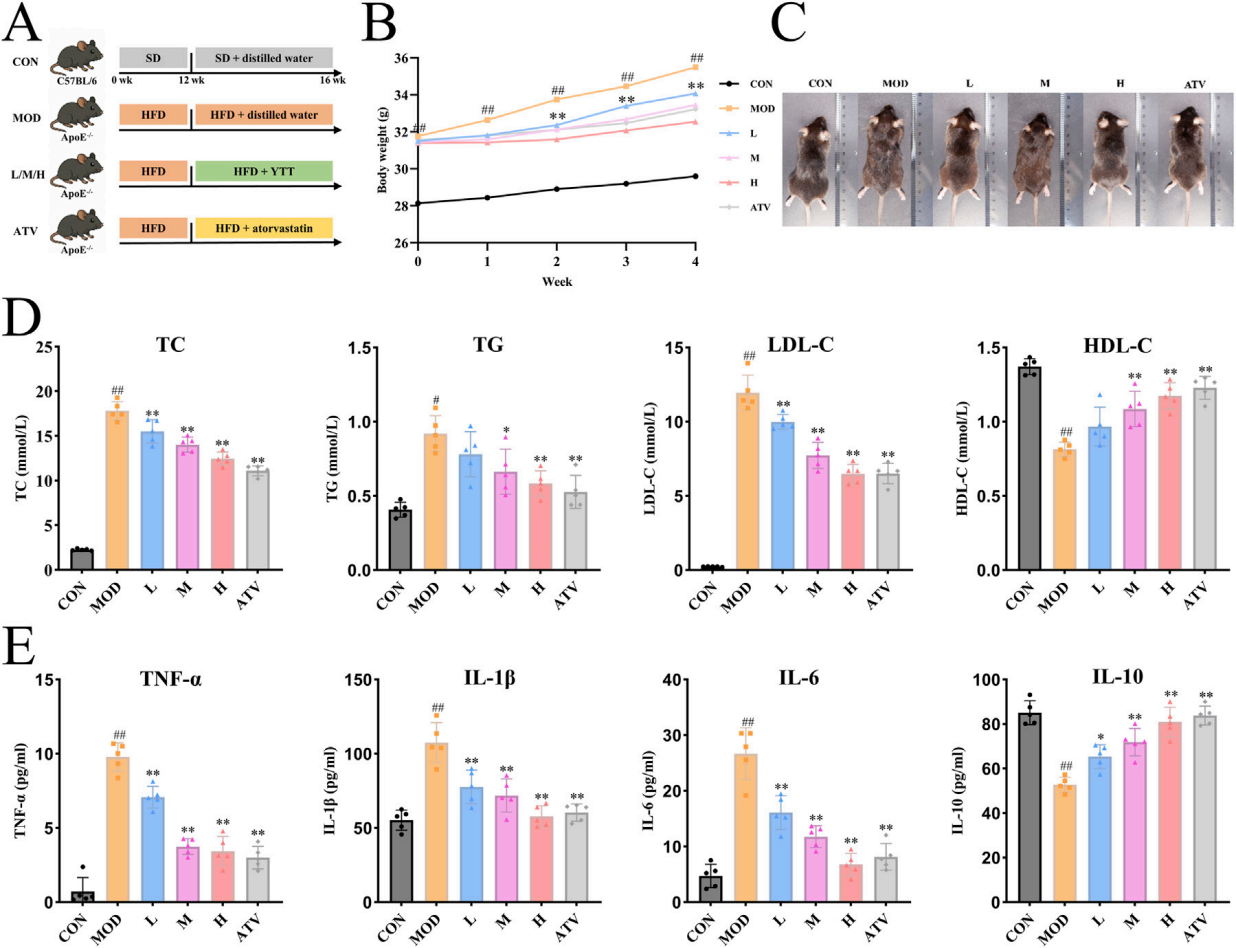
Impact of YTT on Body Weight, Serum Lipid Metabolism, and Inflammatory Cytokine Levels in ApoE^−/−^ Mice Fed a High-Fat Diet. **(A)** Schematic overview of the experimental design. **(B)** Temporal changes in body mass over the intervention period. **(C)** Representative photographs of mice from each experimental group. **(D)** Serum concentrations of TC, TG, LDL-C, and HDL-C (n = 5). **(E)** Serum concentrations of TNF-α, IL-1β, IL-6, and IL-10 (n = 5). Data are expressed as mean ± SD, ^#^ *P* < 0.05, ^##^ *P* < 0.01 vs. control group (CON). *P < 0.05, **P < 0.01 vs. Model group (MOD).

The overall experimental timeline is illustrated in Figure 5A, with representative photographs of mice from each experimental condition provided in Figure 5B. Following 4 weeks of treatment, body weight in the model (MOD) group was significantly higher than that in the control (CON) group (Figure 5C). This increase was markedly reversed after 2 weeks of administration of YTT or atorvastatin. We then analyzed serum lipid metabolism. Notably, MOD group animals developed pronounced dyslipidemia, exhibiting elevated concentrations of TC, TG, and LDL-C, accompanied by reduced HDL-C. YTT treatment at low, medium, and high doses significantly lowered TC, TG, and LDL-C levels, and increased HDL-C concentrations (Figures 5C,D). A similar lipid-modulating effect was observed in the atorvastatin-treated group. Given the established role of chronic inflammation in AS (Bjorkegren and Lusis, 2022), systemic inflammatory markers were also evaluated. Serum concentrations of the pro-inflammatory cytokines TNF-α, IL-1β, and IL-6 were significantly higher in the MOD group relative to the CON group, whereas the anti-inflammatory cytokine IL-10 was considerably reduced (Figure 5E), consistent with a state of persistent low-grade inflammation. Both YTT and atorvastatin significantly attenuated the concentrations of TNF-α, IL-1β, and IL-6, and elevated IL-10 compared to the MOD group.

### 3.6 Impact of YTT on hepatic function

**FIGURE 6:**
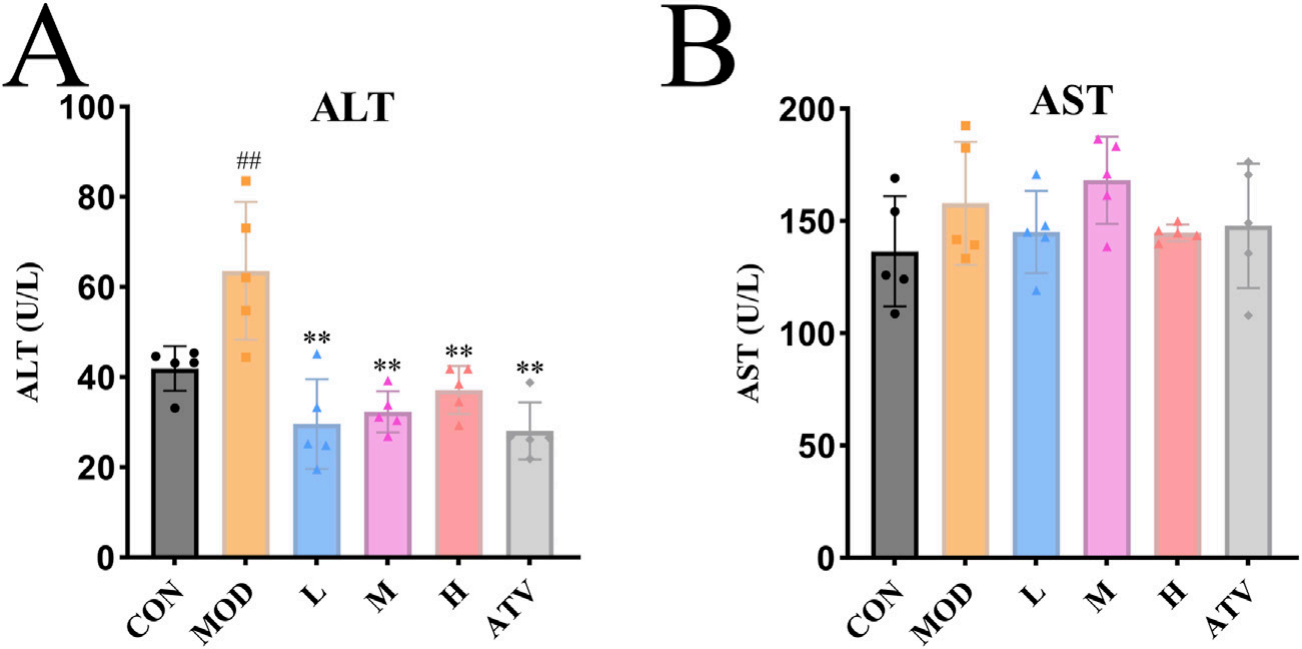
Evaluation of YTT Effects on Hepatic Function. **(A)** Serum ALT concentration (n = 5). **(B)** Serum AST concentration (n = 5). Data are expressed as mean ± SD, ^#^ *P* < 0.05, ^##^ *P* < 0.01 vs. control group (CON). *P < 0.05, **P < 0.01 vs. Model group (MOD).

Serum ALT activity, a sensitive indicator of hepatocellular damage, and AST levels, which may reflect hepatic and cardiac injury, were measured across all groups. As illustrated in Figure 6, the MOD group exhibited a marked elevation in ALT relative to the CON group, suggesting impaired liver function. Administration of YTT at low, medium, and high doses, as well as ATV treatment, significantly attenuated ALT levels compared to the MOD group (Figure 6A), indicating a protective effect of YTT on the liver. In contrast, serum AST levels did not differ significantly among any of the groups (Figure 6B). These results suggest that YTT administration helps restore hepatic function without evidence of hepatotoxicity under the present experimental conditions.

### 3.7 YTT attenuates atherosclerotic lesion development and promotes plaque stability

**FIGURE 7:**
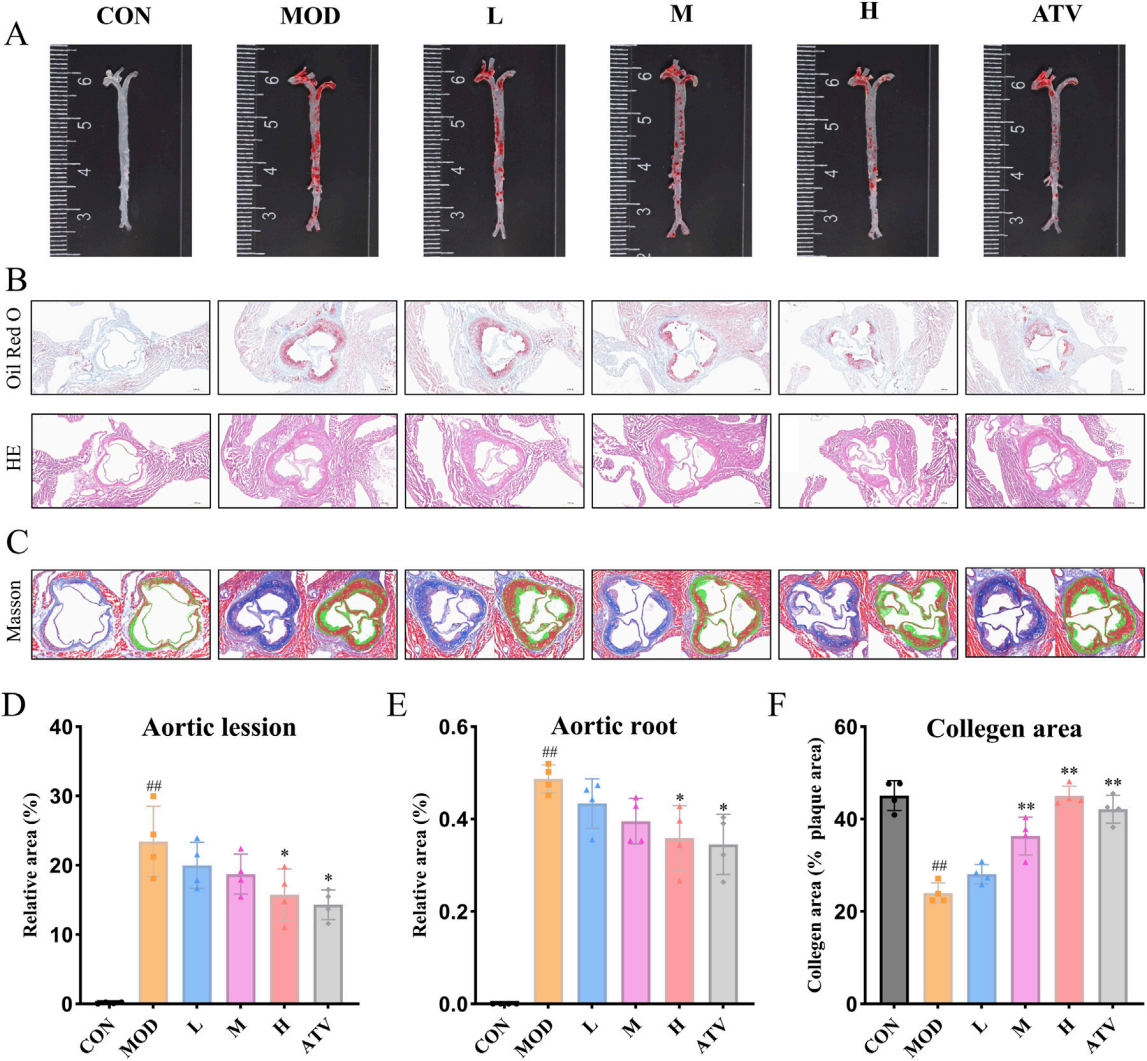
YTT Reduces Atherosclerotic Lesion Development and Promotes Plaque Stability. **(A,D)** Representative *en face* Oil Red O-stained aortae (scale bar: 500 μm) and quantitative analysis of atherosclerotic lesion area (n = 4). **(B,E)** Cross-sections of aortic roots stained with Oil Red O and hematoxylin and eosin **(H,E)** (scale bar: 500 μm), with corresponding quantification of lipid deposition (n = 4). **(C,F)** Masson’s trichrome-stained sections of aortic roots (scale bar: 500 μm) and quantitative assessment of collagen content (n = 4). Data are expressed as mean ± SD, ^#^ *P* < 0.05, ^##^ *P* < 0.01 vs. control group (CON). *P < 0.05, **P < 0.01 vs. Model group (MOD).

To further examine the protective role of YTT in AS, aortic plaque formation and progression were evaluated in ApoE^−/−^ mice. Animals fed a HFD displayed substantial plaque accumulation along with significantly enlarged lipid-rich areas. Administration of YTT across all tested dosages, as well as atorvastatin treatment, markedly reduced both overall aortic plaque burden and lipid deposition (Figures 7A,B,D,E). Plaque stability was assessed via Masson’s trichrome staining of aortic root sections. Compared to the CON group, the MOD group exhibited decreased collagen content within plaques, suggesting increased vulnerability. In contrast, YTT and atorvastatin interventions significantly preserved collagen content in atherosclerotic lesions (Figures 7C,F), indicating enhanced plaque structural integrity. These results demonstrate that YTT not restrains plaque progression but also improves the stability of atherosclerotic lesions *in vivo*.

### 3.8 YTT suppresses activation of the PI3K/Akt/NF-κB pathway in atherosclerosis

**FIGURE 8:**
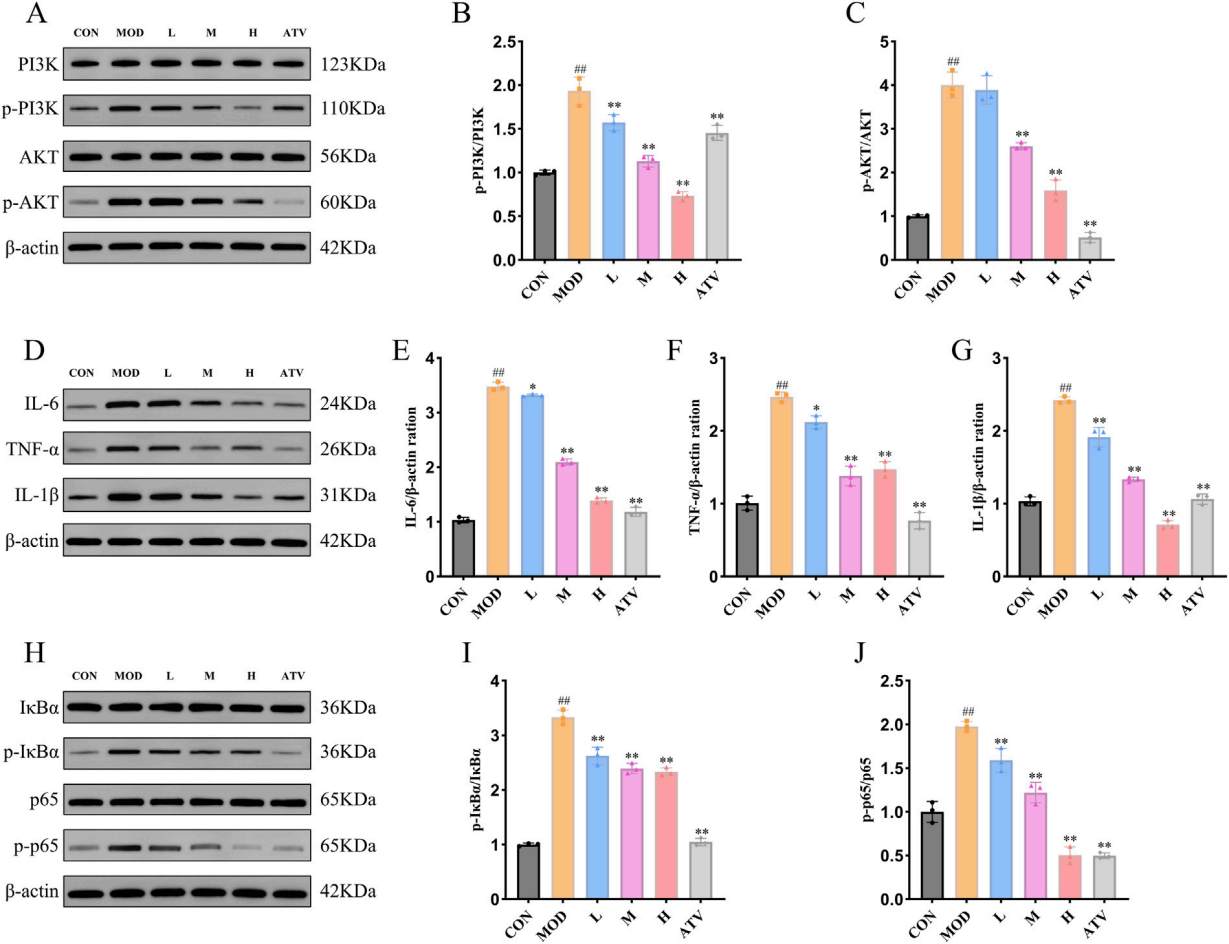
YTT Suppresses Aortic Inflammation via Inhibition of the PI3K/Akt/NF-κB Pathway. **(A–C)** Western blot analysis of PI3K, phospho-PI3K, AKT, and phospho-AKT protein levels in aortic tissue (n = 3). **(D–G)** Western blot evaluation of IL-6, TNF-α, and IL-1β expression in aortic extracts (n = 3). **(H–J)** Western blot detection of IκBα, phospho-IκBα, p65, and phospho-p65 in aortic lysates (n = 3). Data are expressed as mean ± SD, ^#^ *P* < 0.05, ^##^ *P* < 0.01 vs. control group (CON). *P < 0.05, **P < 0.01 vs. Model group (MOD).

Based on network pharmacology, the PI3K-Akt signaling pathway was identified as a candidate mechanism through which YTT might exert its effects in AS. To validate this prediction, we examined the *in vivo* activation status of this pathway. Compared to the CON group, HFD feeding resulted in significantly elevated phosphorylation levels of both PI3K and AKT in aortic tissues (Figures 8A–C). YTT and atorvastatin treatment notably attenuated these phosphorylation events in a dose-dependent manner. Furthermore, AS was associated with increased expression of the pro-inflammatory cytokines TNF-α, IL-1β, and IL-6 in the aorta (Figures 8D–G). YTT administration significantly downregulated these inflammatory mediators, aligning with its predicted anti-inflammatory properties. Given the identification of TNF, IL6, and IL1B as core targets and the established role of PI3K-Akt signaling in inflammatory regulation, we further analyzed the downstream NF-κB pathway, a major effector of inflammatory signaling. The MOD group showed increased phosphorylation of IκBα and p65, indicative of NF-κB pathway activation (Figures 8H–J). YTT treatment effectively suppressed phosphorylation of these proteins, thereby inhibiting NF-κB signaling. These findings indicate that YTT ameliorates atherosclerotic progression by inhibiting the PI3K/Akt/NF-κB axis and subsequent inflammatory responses.

## 4 Discussion

Atherosclerosis (AS) is a chronic inflammatory disease of the vasculature and a primary contributor to cardiovascular and cerebrovascular diseases (Ajoolabady et al., 2024). This study employed an integrated approach, combining network pharmacology, UPLC-QE-MS, molecular docking, and *in vivo* validation, to systematically evaluate the nutraceutical potential and anti-atherosclerotic efficacy of YTT. Administration of YTT was found to significantly decrease levels of pivotal pro-inflammatory cytokines, such as TNF-α, IL-6, and IL-1β, in serum and aortic tissues. Furthermore, it ameliorated disordered lipid metabolism, diminished the overall atherosclerotic plaque area, and promoted plaque stabilization. Mechanistically, the suppression of AS progression by YTT was achieved through the inhibition of the PI3K/Akt/NF-κB signaling pathway, thereby attenuating inflammatory activation. These results substantiate the potential of YTT, a formulation developed based on the FMH concept, as a promising nutraceutical strategy for safeguarding cardiovascular health.

In the network pharmacology, 291 common targets were identified at the intersection of YTT- and AS-associated targets. These targets are primarily associated with essential biological processes, including inflammatory responses and protein phosphorylation. Centrality analysis based on degree further identified TNF, IL6, IL1B, AKT1 as the four most influential hub genes, highlighting the critical involvement of inflammatory signaling in the pathogenesis of AS (Avula et al., 2025; Kong P. et al., 2022; Cao et al., 2024). KEGG enrichment results demonstrated that the common targets participate in pivotal AS-associated pathways, including the PI3K-Akt, Lipid and atherosclerosis, and TNF signaling pathways. Previous reports have emphasized the potential of traditional Chinese medicine to modulate these pathways and ameliorate AS (Li et al., 2025; Liu et al., 2021). As a key downstream mediator of PI3K signaling, AKT1 is abundantly expressed in cardiac, cerebral, and vascular tissues (Fernandez-Hernando et al., 2007) and contributes to the modulation of inflammatory processes (Di Lorenzo et al., 2009). Within the compound-target network, three YTT-derived bioactive compounds—kaempferol, isorhamnetin, and quercetin—were predicted to interact with AKT1. These compounds exhibit established antioxidative, anti-inflammatory, and lipid-modulating activities.

A high-fat dietary regimen reliably elicits dyslipidemia in ApoE^−/−^ mice, an established model for studying AS. Administration of YTT in the current investigation produced significant improvements in systemic lipid parameters, manifesting as reductions in TC, TG, and LDL-C concentrations, accompanied by increased HDL-C. These results corroborate earlier findings on the hypolipidemic properties of FMH constituents in both animal and human studies (Hao et al., 2024), including specific components of YTT such as *Crataegus pinnatifida* (Pang et al., 2021), *Radix Puerariae* (Zhou et al., 2022), and *Panax Ginseng* (Hernandez-Garcia et al., 2019). The pleiotropic nature of FMH-based treatment was further highlighted by its simultaneous impact on lipid homeostasis and inflammatory regulation (Guo H. et al., 2024). Of particular relevance, TNF, IL6, and IL1B were discerned as central hubs within the network; intervention with YTT led to substantial downregulation of these mediators in circulation and aortic tissues, while promoting expression of the anti-inflammatory cytokine IL-10. These outcomes reinforce the well-documented association between disrupted lipid metabolism and the emergence and progression of vascular inflammation (Kong P. et al., 2022). Beyond therapeutic benefits, safety represents an essential requirement for chronic administration in AS management. FMH-derived compounds have demonstrated hepatoprotective properties as nutritional supplements (Deng et al., 2025). In this investigation, animals receiving HFD exhibited increased ALT levels, likely indicative of hepatic stress associated with lipid overload (Longato et al., 2012; Nian et al., 2025), whereas AST levels showed no significant alteration. Notably, both YTT and atorvastatin markedly lowered ALT without elevating AST. Since atorvastatin mitigates HFD-induced hepatic impairment through modulation of lipid metabolism (Sakuma et al., 2025), the liver-protecting activity of YTT may similarly stem from its lipid-regulating capabilities. These collective observations underscore the promise of FMH-based interventions as both safe and efficacious nutritional approaches for the prolonged management of AS.

Advanced atherosclerotic plaques are pathologically defined by an expanded necrotic core (Puylaert et al., 2022), driven primarily by aberrant lipid metabolism and sustained inflammatory activity (Yu et al., 2020). Consistent with biochemical observations, histopathological evaluation in this study demonstrated substantially increased lesion size and extensive lipid accumulation in model group animals. Conversely, YTT treatment resulted in a pronounced reduction in both plaque area and lipid content. Collagen serves as a key structural element of the fibrous cap and is essential for maintaining plaque structural integrity (Di Nubila et al., 2024). Inflammatory mediators promote collagen degradation and compromise plaque stability (Hansson et al., 2015). Notably, YTT intervention significantly enhanced collagen deposition within plaques, indicating a stabilization effect. This finding holds substantial clinical relevance, as vulnerable plaques are susceptible to rupture and may precipitate acute cardiovascular incidents (Kawai et al., 2024).

Mechanistically, our findings strongly support a model in which YTT mediates its anti-atherosclerotic actions, at least partially, through suppression of the PI3K/Akt/NF-κB signaling axis—a well-established regulator of inflammatory responses, lipid homeostasis, and AS development (Liu et al., 2021; Kang et al., 2015). Previous investigations have indicated that extracts derived from FMH alleviates AS by modulating this signaling network (Guo H. et al., 2024). For instance, Luo et al. demonstrated that the flavonoid isorhamnetin activates PI3K/Akt signaling, diminishing reactive oxygen species generation and lipid accumulation, thereby limiting plaque development (Luo et al., 2015). In contrast, sustained activation of PI3K/Akt may potentiate inflammatory processes by enhancing immune cell infiltration and pro-inflammatory cytokine secretion (Feng et al., 2023; Morello et al., 2009). It is noteworthy that PI3K/Akt/NF-κB signaling is frequently upregulated during advanced stages of AS, where it predominantly exerts pro-inflammatory effects (Guo Q. et al., 2024). In the present study, YTT supplementation markedly inhibited phosphorylation of both PI3K and AKT, consistent with attenuation of this pathway. As a principal downstream effector, NF-κB critically governs transcriptional induction of multiple pro-inflammatory cytokines (Guo H. et al., 2024; Kong X. et al., 2022). Kang et al. reported that NF-κB activation strongly correlates with vascular inflammation in HFD-fed ApoE^−/−^ mice (Kang et al., 2015). Concordant with these reports, we found that YTT decreased phospho-NF-κB levels and substantially reduced TNF-α, IL-6, and IL-1β expression in aortic tissues.

Notwithstanding the encouraging results, several limitations of this study warrant consideration. First, the network pharmacology predictions were derived from human protein databases, whereas the experimental validation was conducted in a murine model. Although the core pathophysiology of atherosclerosis is highly converved, clinical trials are therefore essential to verify the effectiveness and safety of YTT in human subjects and validate the involvement of the identified targets in human patients. Second, the mechanistic exploration concentrated primarily on a single signaling cascade. Owing to the multi-target characteristics of FMH-based formulations, subsequent research should investigate other molecular pathways, especially those related to lipid metabolism and immunomodulation. Third, although network pharmacology and molecular docking offered useful predictive information, this study did not perform extensive protocol validation or complementary docking metrics. In future studies, we plan to strengthen this aspect by incorporating more advanced molecular docking strategies and machine learning–based prediction or scoring approaches, together with experimental verification—such as through gene knockdown, overexpression, or selective pathway inhibition—is required to substantiate the roles of the identified targets. We also acknowledge that we did not directly measure plasma concentrations of the individual YTT components in the present experiment. Future pharmacokinetic studies are therefore needed to characterize systemic exposure to the major active constituents (e.g., kaempferol, isorhamnetin, quercetin) and to more precisely define the relationship between dose, plasma levels, and anti-atherosclerotic efficacy. These efforts will better frame the dose selection and translational implications of our *in vivo* findings. Finally, given the inherent variability in the composition of herbal formulations, follow-up studies should include quantitative assessment of compound levels and verification of the *in vivo* bioavailability of active ingredients.

## 5 Conclusion

In summary, YTT intervention significantly improved dyslipidemia, attenuated inflammation, and ameliorated atherosclerotic plaque formation in experimental models of AS. Through an integrated approach combining network pharmacology and molecular docking, kaempferol, isorhamnetin, and quercetin were identified as primary bioactive compounds, likely mediating their effects via suppression of the PI3K/Akt/NF-κB pathway. These results underscore the importance of inflammatory regulation in the vasculoprotective actions of YTT. Future research should focus on advanced modeling techniques such as human vascular or plaque organoid systems to better mimic the pathophysiology of AS and validate the effects of YTT/AKT1. Additionally, CRISPR-based perturbation of the PI3K/Akt/NF-κB pathway could be used to validate the target dependence and explore the translational potential of YTT in clinical settings. More broadly, this work supports the further development of FMH-based formulations as complementary strategies for preventing and managing atherosclerosis.

## Data Availability

The original contributions presented in the study are included in the article/Supplementary Material, further inquiries can be directed to the corresponding authors.

## References

[B1] Abulaiti K. Aikepa M. Ainaidu M. Wang J. Yizibula M. Aikemu M. (2024). Metabolomics combined with network pharmacology reveals anti-asthmatic effects of Nepeta bracteata on allergic asthma rats. Chin. Herb. Med. 16, 599–611. 10.1016/j.chmed.2024.02.001 39606263 PMC11589474

[B2] Ajoolabady A. Pratico D. Lin L. Mantzoros C. S. Bahijri S. Tuomilehto J. (2024). Inflammation in atherosclerosis: pathophysiology and mechanisms. Cell Death Dis. 15, 817. 10.1038/s41419-024-07166-8 39528464 PMC11555284

[B3] Avula V. Mok Y. Ejiri K. Van't Hof J. Whelton S. P. Hoogeveen R. C. (2025). Inflammatory markers and calcification of coronary arteries, aorta and cardiac valves: findings from the atherosclerosis risk in communities study. Am. J. Prev. Cardiol. 21, 100946. 10.1016/j.ajpc.2025.100946 40060173 PMC11889617

[B4] Bjorkegren J. L. M. Lusis A. J. (2022). Atherosclerosis: recent developments. Cell 185, 1630–1645. 10.1016/j.cell.2022.04.004 35504280 PMC9119695

[B5] Cao W. Wang K. Wang J. Chen Y. Gong H. Xiao L. (2024). Causal relationship between immune cells and risk of myocardial infarction: evidence from a Mendelian randomization study. Front. Cardiovasc Med. 11, 1416112. 10.3389/fcvm.2024.1416112 39257847 PMC11384581

[B6] Chen Y. Yang L. Wang K. An Y. Wang Y. Zheng Y. (2024). Relationship between fatty acid intake and aging: a Mendelian randomization study. Aging (Albany NY) 16, 5711–5739. 10.18632/aging.205674 38535988 PMC11006485

[B7] Deng Y. Cui J. Jiang Y. Zhang J. Jiang J. Zhang Q. (2025). Exploring the nutraceutical potential of a food-medicine compound for metabolic-associated fatty liver disease via lipidomics and network pharmacology. Foods 14, 1257. 10.3390/foods14071257 40238509 PMC11988326

[B8] Di Lorenzo A. Fernandez-Hernando C. Cirino G. Sessa W. C. (2009). Akt1 is critical for acute inflammation and histamine-mediated vascular leakage. Proc. Natl. Acad. Sci. U. S. A. 106, 14552–14557. 10.1073/pnas.0904073106 19622728 PMC2732859

[B9] Di Nubila A. Dilella G. Simone R. Barbieri S. S. (2024). Vascular extracellular matrix in atherosclerosis. Int. J. Mol. Sci. 25, 12017. 10.3390/ijms252212017 39596083 PMC11594217

[B10] Feng X. Du M. Li S. Zhang Y. Ding J. Wang J. (2023). Hydroxysafflor yellow A regulates lymphangiogenesis and inflammation via the inhibition of PI3K on regulating AKT/mTOR and NF-κB pathway in macrophages to reduce atherosclerosis in ApoE-/- mice. Phytomedicine 112, 154684. 10.1016/j.phymed.2023.154684 36738477

[B11] Fernandez-Hernando C. Ackah E. Yu J. Suarez Y. Murata T. Iwakiri Y. (2007). Loss of Akt1 leads to severe atherosclerosis and occlusive coronary artery disease. Cell Metab. 6, 446–457. 10.1016/j.cmet.2007.10.007 18054314 PMC3621848

[B12] Gao H. Kang N. Hu C. Zhang Z. Xu Q. Liu Y. (2020). Ginsenoside Rb1 exerts anti-inflammatory effects *in vitro* and *in vivo* by modulating toll-like receptor 4 dimerization and NF-kB/MAPKs signaling pathways. Phytomedicine 69, 153197. 10.1016/j.phymed.2020.153197 32146298

[B13] Guo H. Cui B. D. Gong M. Li Q. X. Zhang L. X. Chen J. L. (2024). An ethanolic extract of Arctium lappa L. leaves ameliorates experimental atherosclerosis by modulating lipid metabolism and inflammatory responses through PI3K/Akt and NF-κB singnaling pathways. J. Ethnopharmacol. 325, 117768. 10.1016/j.jep.2024.117768 38253275

[B14] Guo Q. Jin Y. Chen X. Ye X. Shen X. Lin M. (2024). NF-κB in biology and targeted therapy: new insights and translational implications. Signal Transduct. Target Ther. 9, 53. 10.1038/s41392-024-01757-9 38433280 PMC10910037

[B15] Hansson G. K. Libby P. Tabas I. (2015). Inflammation and plaque vulnerability. J. Intern Med. 278, 483–493. 10.1111/joim.12406 26260307 PMC5082111

[B16] Hao X.-T. Peng R. Guan M. Zhang H.-J. Guo Y. Shalapy N. M. (2024). The food and medicinal homological resources benefiting patients with hyperlipidemia: categories, functional components, and mechanisms. *Food and Med. Homol.* 1, 9420003. 10.26599/fmh.2024.9420003

[B17] Hernandez-Garcia D. Granado-Serrano A. B. Martin-Gari M. Naudi A. Serrano J. C. (2019). Efficacy of Panax ginseng supplementation on blood lipid profile. A meta-analysis and systematic review of clinical randomized trials. J. Ethnopharmacol. 243, 112090. 10.1016/j.jep.2019.112090 31315027

[B18] Huang F. Mu J. Liu Z. Lin Q. Fang Y. Liang Y. (2023). The nutritional intervention of ingredients from food medicine homology regulating macrophage polarization on atherosclerosis. J. Agric. Food Chem. 71, 20441–20452. 10.1021/acs.jafc.3c06375 38108290

[B19] Kang Q. Liu W. Liu H. Zhou M. (2015). Effect of compound chuanxiong capsule on inflammatory reaction and PI3K/Akt/NF-κB signaling pathway in atherosclerosis. Evid. Based Complement. Altern. Med. 2015, 584596. 10.1155/2015/584596 26539229 PMC4619937

[B20] Kawai K. Kawakami R. Finn A. V. Virmani R. (2024). Differences in stable and unstable atherosclerotic plaque. Arterioscler. Thromb. Vasc. Biol. 44, 1474–1484. 10.1161/ATVBAHA.124.319396 38924440

[B21] Kong P. Cui Z. Y. Huang X. F. Zhang D. D. Guo R. J. Han M. (2022). Inflammation and atherosclerosis: signaling pathways and therapeutic intervention. Signal Transduct. Target Ther. 7, 131. 10.1038/s41392-022-00955-7 35459215 PMC9033871

[B22] Kong X. Chen H. Li D. Ma L. (2022). Effects of imbalance of lipid metabolism through NF-KB pathway on atherosclerosis and vascular aging in rats. Cell Mol. Biol. (Noisy-le-grand) 67, 144–150. 10.14715/cmb/2021.67.5.20 35818259

[B23] Li Z. Weng J. Yan J. Zeng Y. Hao Q. Sheng H. (2024). Puerarin alleviates atherosclerosis via the inhibition of Prevotella copri and its trimethylamine production. Gut 73, 1934–1943. 10.1136/gutjnl-2024-331880 38777572

[B24] Li H. Yang W. Cao W. Yu Z. Zhang G. Long L. (2024). Effects and mechanism of kedaling tablets for atherosclerosis treatment based on network pharmacology, molecular docking and experimental study. J. Ethnopharmacol. 319, 117108. 10.1016/j.jep.2023.117108 37657772

[B25] Li X. Ding L. Li Z. Cao Z. Li M. Yin K. (2025). Yangke powder alleviates OVA-induced allergic asthma by inhibiting the PI3K/AKT/NF-κB signaling pathway. Chin. Med. 20, 69. 10.1186/s13020-025-01125-x 40420184 PMC12105270

[B26] Lin J. Wang X. Gu M. Chen Y. Xu J. Chau N. V. (2024). Geniposide ameliorates atherosclerosis by restoring lipophagy via suppressing PARP1/PI3K/AKT signaling pathway. Phytomedicine 129, 155617. 10.1016/j.phymed.2024.155617 38614041

[B43] Liu H. Wang J. Zhou W. Wang Y. Yang L. (2013). Systems approaches and polypharmacology for drug discovery from herbal medicines: an example using licorice. J. Ethnopharmacol. 146, 773–793. 10.1016/j.jep.2013.02.004 23415946

[B27] Liu J. Xu P. Liu D. Wang R. Cui S. Zhang Q. (2021). TCM regulates PI3K/Akt signal pathway to intervene atherosclerotic cardiovascular disease. Evid. Based Complement. Altern. Med. 2021, 4854755. 10.1155/2021/4854755 34956379 PMC8702326

[B28] Longato L. Tong M. Wands J. R. de la Monte S. M. (2012). High fat diet induced hepatic steatosis and insulin resistance: role of dysregulated ceramide metabolism. Hepatol. Res. 42, 412–427. 10.1111/j.1872-034X.2011.00934.x 22176347 PMC4096625

[B29] Luo Y. Sun G. Dong X. Wang M. Qin M. Yu Y. (2015). Isorhamnetin attenuates atherosclerosis by inhibiting macrophage apoptosis via PI3K/AKT activation and HO-1 induction. PLoS One 10, e0120259. 10.1371/journal.pone.0120259 25799286 PMC4370599

[B30] Mensah G. A. Fuster V. Murray C. J. L. Roth G. A. Global Burden of Cardiovascular Diseases and Risks Collaborators (2023). Global burden of cardiovascular D, risks C: Glogal burden of cardiovascular diseases and risks, 1990-2022. J. Am. Coll. Cardiol. 82, 2350–2473. 10.1016/j.jacc.2023.11.007 38092509 PMC7615984

[B31] Morello F. Perino A. Hirsch E. (2009). Phosphoinositide 3-kinase signalling in the vascular system. Cardiovasc Res. 82, 261–271. 10.1093/cvr/cvn325 19038971

[B32] Nian S. Wang K. Wang J. Wang S. Li C. Li N. (2025). Causal associations between immune cell phenotypes and varicose veins: a mendelian randomization analysis. Ann. Vasc. Surg. 114, 126–132. 10.1016/j.avsg.2025.01.030 39884498

[B33] Pang X. Wang M. Wang S. Y. Zhang J. Du Y. P. Zhao Y. (2021). Phenolic compounds from the leaves of Crataegus pinnatifida Bge. var. major N.E.Br. And their lipid-lowering effects. Bioorg Med. Chem. Lett. 47, 128211. 10.1016/j.bmcl.2021.128211 34157392

[B34] Puylaert P. Zurek M. Rayner K. J. De Meyer G. R. Y. Martinet W. (2022). Regulated necrosis in atherosclerosis. Arterioscler. Thromb. Vasc. Biol. 42, 1283–1306. 10.1161/ATVBAHA.122.318177 36134566

[B35] Ramkumar S. Raghunath A. Raghunath S. (2016). Statin therapy: review of safety and potential side effects. Acta Cardiol. Sin. 32, 631–639. 10.6515/acs20160611a 27899849 PMC5126440

[B36] Riccardi G. Giosue A. Calabrese I. Vaccaro O. (2022). Dietary recommendations for prevention of atherosclerosis. Cardiovasc Res. 118, 1188–1204. 10.1093/cvr/cvab173 34229346

[B37] Sakuma I. Gaspar R. C. Nasiri A. R. Dufour S. Kahn M. Zheng J. (2025). Liver lipid droplet cholesterol content is a key determinant of metabolic dysfunction-associated steatohepatitis. Proc. Natl. Acad. Sci. U. S. A. 122, e2502978122. 10.1073/pnas.2502978122 40310463 PMC12067271

[B38] Vesnina A. Prosekov A. Atuchin V. Minina V. Ponasenko A. (2022). Tackling atherosclerosis via selected nutrition. Int. J. Mol. Sci. 23, 8233. 10.3390/ijms23158233 35897799 PMC9368664

[B39] Wang Y. Li Z. He J. Zhao Y. (2024). Quercetin regulates lipid metabolism and fat accumulation by regulating inflammatory responses and glycometabolism pathways: a review. Nutrients 16, 1102. 10.3390/nu16081102 38674793 PMC11053503

[B40] Wang L. Zhu X. Liu H. Sun B. (2025). Medicine and food homology substances: a review of bioactive ingredients, pharmacological effects and applications. Food Chem. 463, 141111. 10.1016/j.foodchem.2024.141111 39260169

[B41] Yu X. H. Chen J. J. Deng W. Y. Xu X. D. Liu Q. X. Shi M. W. (2020). Biochanin A mitigates atherosclerosis by inhibiting lipid accumulation and inflammatory response. Oxid. Med. Cell Longev. 2020, 8965047. 10.1155/2020/8965047 33959213 PMC8074550

[B42] Zhou X. Yu J. Wan Q. Wang W. Yu X. You J. (2022). Efficacy and safety of Pueraria lobata radix and Pueraria thomsonii radix for patients with mild dyslipidemia: a randomized, double-blind, placebo-controlled trial. J. Funct. Foods 98, 105284. 10.1016/j.jff.2022.105284

